# Mechanical and thermal efficiency of a single drill system for bone-anchored hearing implants

**DOI:** 10.1371/journal.pone.0311026

**Published:** 2025-05-30

**Authors:** Marsel Ganeyev, Furqan A. Shah, Peter Thomsen, Anders Palmquist, Martin L. Johansson

**Affiliations:** 1 Department of Biomaterials, Institute of Clinical Sciences, Sahlgrenska Academy, University of Gothenburg, Gothenburg, Sweden; 2 Oticon Medical AB, Askim, Sweden; University of Vigo, SPAIN

## Abstract

**Purpose:**

This study aimed to evaluate the mechanical performance, heat generation, bone distortion, and characteristics of bone chips generated during drilling using a novel one-step guided drill system (MONO) for installing the bone-anchored hearing system (BAHS). A comparison was made with an existing three-stage drill system (MIPS).

**Materials and Methods:**

Drill force and torque were measured during drilling in cow tibia at different feed rates. Compact artificial bone was utilized to determine temperature increases using thermocouples placed at specific positions around the osteotomy site during drilling with the two systems at different feed rates and levels of irrigation. The effects of drilling on osteotomy characteristics and the formation of bone fragments were evaluated through micro-CT, Raman spectroscopy, and histology.

**Results:**

Force and torque increased with the feed rate in both systems, whereas the total work required to perform the osteotomy significantly decreased as the feed rate increased. Compared to the three-stage MIPS system, the MONO system required less work for one-step osteotomy creation, generated equal or less heat, and exhibited greater tolerance for procedural deviations in irrigation and drilling sequence. Additionally, heat generation for both systems decreased when drilling at higher feed rates. Compositional changes within the osteotomy were primarily observed under reduced irrigation protocols, while no differences were identified in bone chips across drilling protocols.

**Conclusion:**

Compared with a multistep conventional drilling procedure, MONO drilling is less affected by variations in the drilling protocol, particularly in flapless and blind procedures, resulting in safer and more efficient osteotomy creation. The MONO system demonstrated superior performance in terms of energy efficiency and temperature control.

## Introduction

There is a wide range of applications for bone-anchored, percutaneous implants, including within orthopedics, dentistry and hearing rehabilitation. Various strategies have been implemented to enhance tissue integration and expedite osseointegration. These approaches involve the modification of implant designs and the application of diverse surface modification strategies. [1,2]. However, it is crucial to acknowledge that the surgical procedure for generating the osteotomy is also an important component for subsequent successful osseointegration of the implant.

Typically, osteotomy is generated using a rotating metal bur. The heat generated during this procedure may, however, harm the surrounding bone and potentially affect subsequent healing and osseointegration [3–6]. The two main strategies to mitigate this risk for overheating are the use of irrigation to cool the drilling site and the use of a multistep drilling protocol involving sequential enlargement and deepening of the osteotomy site. Multiple other factors during site preparation may affect, alone or in combination, heat generation and the quality of the osteotomy, including drill depth, drilling duration, force applied during drilling [7], rotational speed [8], drill bit design (e.g., flute design, cutting properties, material, surface characteristics) [9], wear of drill [10], hardness of the bone [11], drilling protocol (single step or incremental, guided or nonguided) [12,13] and type of irrigation (internal, external, temperature of irrigant) [14,15]. Additionally, operators play a significant role, as their experience, skill, and technique directly impact the precision, safety, and effectiveness of the drilling process [3–6].

The percutaneous bone-anchored hearing system (BAHS), which is used for treating conductive or mixed hearing loss [16], consists of a screw-shaped titanium implant mounted with an abutment to which a sound processor is connected. The implant is inserted into the temporal bone in a retroauricular position, leaving the abutment permanently protruding through the scalp to allow for the attachment of the sound processor. BAHS improves hearing ability as well as quality of life [17] and is associated with a survival rate of up to 98%, particularly when newer generation wide diameter implants and surface modifications are used [17–19]. However, it can also lead to several complications, including adverse soft tissue reactions, pain, and aesthetic implications [20]. The surgical procedure of BAHSs has traditionally utilized a linear incision approach together with a three-step drilling protocol for the preparation of the bone bed prior to installation of the implant [21,22]. Recently, minimally invasive site preparation was introduced, wherein the drilling procedure was performed via a guide inserted in a circular biopsy incision in the skin [23,24]. This technique, minimally invasive Ponto surgery (MIPS) (Oticon Medical, AB, Askim, Sweden), also uses a three-step drilling approach with sequential enlargement and deepening of the implant site.

Despite successful clinical outcomes, this approach may be associated with potential drawbacks compared with the open linear incision method, where the bone site remains exposed and visible during site preparation. Like flapless dental implant surgery, challenges with guided drilling procedures include the need for appropriate training, as there appears to be a learning curve to achieve treatment success using flapless implant placement [3–6]; possible complications, such as the inclusion of soft tissue in the osteotomy [3,25,26]; and reduced surgical access, leading to a risk of inadequate irrigation, potentially creating heat-induced necrosis of the bone [27–31]. However, the use of the MIPS procedure for the implantation of BAHSs has provided notable benefits for patients compared with previously utilized surgical methods. Clinical studies have indicated a significant reduction in the duration of surgery, comparable or improved conditions of the surrounding soft tissues, better cosmetic outcomes, and decreased numbness around the abutment [23,32,33]. Furthermore, evaluations in artificial bone revealed that the MIPS drilling protocol provided more efficient osteotomy site preparation but resulted in greater heat generation than did the conventional drilling procedure; however, the temperatures recorded were below the threshold of causing bone damage [24]. In 2018, several changes were introduced to the original design of the MIPS drill, as the first version was sensitive to deviations in the protocol, leading to possible complications [12,32]. To address the potential limitations of the multidrill guided approach employed in MIPS, a new parabolic drill (MONO) was developed. This drill aims to enhance the cutting capability, facilitating one-step preparation of a 4 mm BAHS implant site while minimizing the risk of mechanical and thermal injury. Therefore, to fully understand the clinical performance of the MIPS and MONO systems, a comparative evaluation is essential to assess their mechanical and thermal performance, effects on osteotomy shape and bone composition, and sensitivity to protocol deviations.

The influence of drill design and drilling protocol on heat generation is typically studied using thermocouples, infrared temperature measurements and FEM simulations, each of which have benefits and drawbacks [34]. Many studies have evaluated the effects of the drilling speed, feed rate, drill design, substrate, and cooling on heat generation during implant site preparation [13,35–37]. However, the lack of consistency in methodology makes it challenging to extract the optimal drill design and drilling protocol from the collected data and apply it to a specific system (in terms of implant design, drill protocol and design, location and patient characteristics). Additionally, even though there is a dogma that specifies 47°C [28,38] as a limiting temperature when the drilling duration is > 1 min [39], there are additional factors that come into play, such as total work applied, drill design, and the presence of irrigation [40]. Another significant aspect is the potential role of bone debris generated during drilling in the process of implant osseointegration [35,41]. Although heat from drilling can cause bone decomposition to occur as a result of osteonecrosis during osteotomy [42], research investigating its effects is limited. While some studies have proposed that these autologous bone particles can play a positive role in bone osseointegration [41], they are regularly removed prior to implant installation [43]. The purpose of this experimental bench and *ex vivo* study was to compare the use of a new flapless single-step drill system (MONO) with that of a clinically used 3-step flapless drill system (MIPS) in terms of mechanical and thermal performance, osteotomy and bone fragment characteristics, and the influence of irrigation and drilling duration.

## Materials and methods

### Drill system

**Fig 1:**
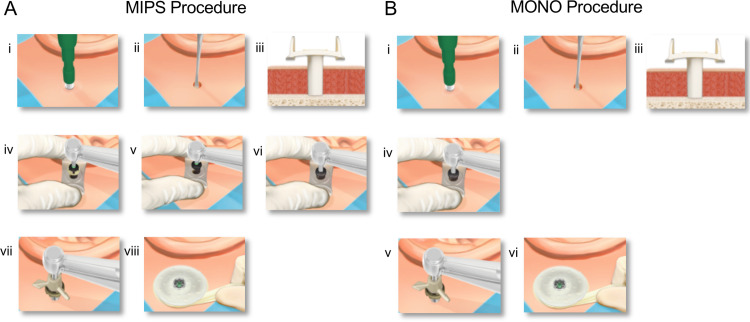
(A) Procedural steps for minimally invasive Ponto surgery (MIPS). The MIPS procedure involves the following steps: (i) A circular incision is created at the selected site via a 4- or 5-mm biopsy punch. (ii) The periosteum around the surgical site is removed. (iii) The cannula is inserted into the incision. (iv) First, initial guide drilling is performed. (v) If the bone thickness is adequate, the spacer should be removed from the guide drill to prepare for a 4-mm implant. (vi) The hole is widened with a widening drill. (vii) The cannula is removed, and the implant is installed through the circular incision. (viii) Finally, a soft healing cap is attached to the abutment, and a dressing is applied. (B) The surgical steps for the MONO procedure. The MONO procedure involves the following steps: (i) Create a circular incision at the chosen site via a 4- or 5-mm biopsy punch. (ii) The periosteum around the surgical site is removed. (iii) The cannula is inserted into the incision. (iv) The final osteotomy is generated in a single step via the MONO drill. (vii) The cannula is removed, and the implant is installed through the circular incision. (viii) Finally, a soft healing cap is attached to the abutment, and a dressing is applied. Images used with permission obtained from Oticon Medical AB©.

Traditionally, for installation of the BAHS, a linear incision exposing the bone surface was used prior to performing the 3-step drilling procedure to generate the osteotomy for placement of the BAHS implant (4 mm long, 4.5 mm diameter) [44]. A decade ago, a flapless, guided surgical procedure, the minimally invasive Ponto system (MIPS), was introduced, mimicking the traditional 3-step drilling sequence but with the difference that drilling is performed via a cannula inserted in a punch incision in the skin [24,44,45] (Fig 1A).

More recently, a new guided drill system (MONO) was implemented, which uses a minimally invasive approach via the use of a cannula (Fig 1B). In the MONO procedure, however, the osteotomy is created using one drilling step in contrast to the three-step drilling procedure used for MIPS (Fig 1A-B). Whereas the MIPS drill bits follow a conventional twist drill design, the MONO drill has a parabolic design. This feature minimizes the amount of metal in the drill bit and hence the amount of metal in contact with the surrounding bone, potentially leading to less friction and heat generation for the MONO drill than for the two MIPS drill holes. Importantly, this approach also substantially increases the space available for irrigants while facilitating the efficient removal of hot bone fragments from the osteotomy site.

**Fig 2:**
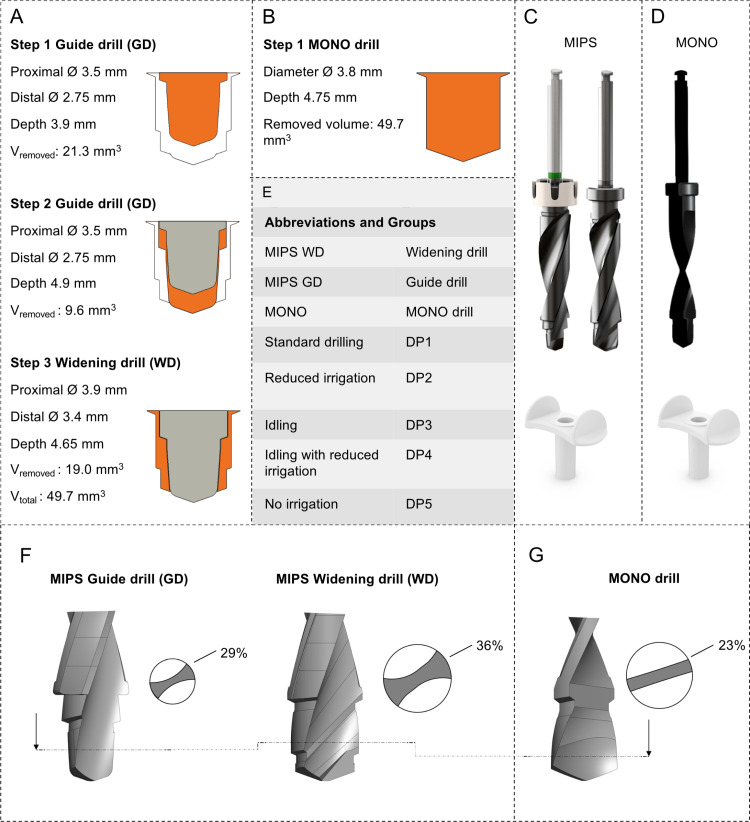
(A) Shape of the osteotomies during the 3-step MIPS drilling procedure. Initially, a 3.9-mm deep hole is crafted using the guide drill (step 1), followed by deepening it by an additional millimetre to accommodate a 4-mm implant (step 2). Ultimately, the osteotomy is expanded via a widening drill for the guided system (step 3). (B) A one-step MIPS drilling procedure, where osteotomy is created in one step, is shown. (C) The MIPS drill system with a guide drill (GD) and widening drill (WD). (D) The MONO drill system, consisting of the MONO drill (top) and the cannula (below). (E) Overview of the abbreviations employed for the drill systems and protocols (DP1-DP5). (F-G) displays the drill bit designs, including a cross-sectional representation indicating the percentage of area occupied by the drill bit. Panels C-G: Images used with permission obtained from Oticon Medical AB©.

In comparison, the MONO drill accounts for 23% of the cross-sectional area in the osteotomy area, whereas the corresponding areas are 29% and 36% for the MIPS GD and MIPS WD, respectively (Fig 2F, G). Moreover, compared with those of conventional twist drills, the parabolic shape of the cutting edges of drill bits allows them to be designed with increased bone cutting capability. Taken together, these findings lead us to hypothesize that these features enable the generation of an osteotomy for a 4 mm long BAHS implant in one single drill step without requiring excessive force or the risk of thermal injury to the surrounding bone.

All drill bits were made of stainless steel and treated with a diamond-like carbon coating. All the drills, tools, and testing materials used were supplied by the manufacturer Oticon Medical (Askim, Sweden).

### *Ex vivo* mechanical evaluation

The cutting characteristics of the three drill bits were determined by measuring the torque and force using a specially designed test rig (Asset No. A069, Oticon Medical)**,** presented in S1 Fig, while drilling in fresh cadaveric cow tibia bone from a local butcher. Drilling was performed at three different feed rates (0.5, 1.0 and 2 mm/sec) and a constant rotational speed of 2,000 rpm. For technical reasons related to the control of the rig, the first two drilling steps in the MIPS procedure (MIPS Guide Drill) were performed to full depth (4.9 mm) in one step, as opposed to the clinical situation where the guide hole is generated in a two-step sequence (Fig 2). Five drilling sequences were recorded for two individual sets of drills, resulting in a total of 10 measurements for each combination of drill, substrate, and feed rate. All drill sequences were performed via the cannula without irrigation at an ambient temperature of 22 ± 2 °C. Using the recorded force and torque data, the power was calculated according to the following equations:


$$P_{F}=F*MPS$$

$$P_{T}=2\pi *n*T$$

Here, P_F_ is the thrust power, F is the force in Newtons, MPS is the feed rate in meters per second, P_T_ is the power from the torque, and n is the number of rotations per second. The total amount of thrust and torque work for the drill sequences was obtained by calculating the area under the curves (Microsoft Excel, v16.80; Microsoft, USA).

### *In vitro* heat generation

**Fig 3:**
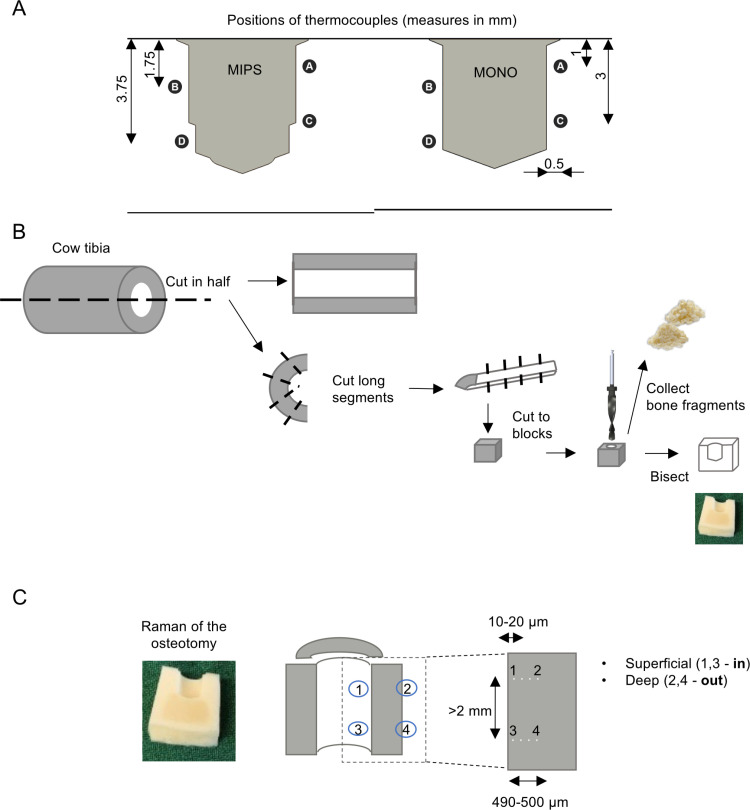
(A) Thermocouple positions for the MIPS and MONO systems. (B) Preparation of bone cubes from fresh cow tibias further employed for ex vivo mechanical evaluation, osteotomy and bone fragment analysis. (C) Overview of the reference points at which the Raman spectra were obtained; (1) and (3) are the points in proximity to the osteotomy site, and (2) and (4) are the outer points further away from the osteotomy site.

The test was designed to measure the temperature increase around the osteotomy site during site preparation for a 4 mm long implant with a diameter of 4.5 mm (Ponto Wide 4 mm, Oticon Medical AB, Sweden), thereby simulating the clinical procedure. Site preparation was performed in artificial bone blocks (Sawbones PCF 50, REF 1522–27, Sawbones Europe AB, Limhamn, Sweden) [46] using either the MIPS three-step drilling sequence or the MONO one-step drilling sequence. During drilling, the temperature increase was recorded at four different depths around the osteotomy site using thermocouples (type K, Model 363–0250, range −75 °C to 250 °C, accuracy according to IEC-584-3 Class 1, RS Components, Gothenburg, Sweden) connected to a data logger (sampling rate 10 measurements per second, TC-08 Data logger, PICO, Cambridgeshire, UK) (Fig 3A). To ascertain the position of the thermocouples, artificial bone blocks were prepared with four canals for housing the thermocouples, leaving the tip of the thermocouple 0.5 mm from the final periphery of the osteotomy. The thermocouples were secured, and the canal openings were sealed with adhesive (Tack-It, Faber-Castell, Stein, Germany). Freehand drilling was performed by an experienced operator using a surgical drill unit and handpiece (Implantmed SI-923 Dental drill unit, Handpiece WI-75E/KM 20:1, W&H Nordic, Täby, Sweden) with a drilling speed set at 2,000 rpm. Five distinct drilling procedures were defined (DP1-DP5) to simulate various clinical scenarios in the assumed order of increased heat generation resulting from deviations from the recommended standard procedure in terms of irrigation efficiency and duration of the drilling sequence.

**DP1**. The samples were drilled according to the recommended standard procedure with irrigation provided. Here, tap water (22 °C) was manually perfused with a 20-cc syringe. The cannula was prefilled with water before the drill was inserted, with continuous irrigation during each drilling sequence and flushing of the osteotomy after the drill bit was removed from the site.
**DP2**. Like in DP1, the cannula is filled with water prior to each drill step, but continuous irrigation during the drilling and flushing of the osteotomy after drilling are not conducted.
**DP3**. Like in DP1, but additionally each drill is left idling during the osteotomy (rotating at 2,000 rpm) for approximately two seconds after it reaches its full depth.
**DP4**. Combination of idling (DP2) and reduced irrigation (DP3).
**DP5**: As a final worst-case (positive control) condition, drilling is performed without any irrigation.

In total, ten osteotomies were prepared for each combination of system (MIPS, MONO) and drilling procedure (DP1-DP5). After being used five times, each drill bit was replaced with a new, unused drill.

In addition to the manual site preparations, the influence of the feed rate on heat generation was evaluated by using the same test rig used for the mechanical evaluation (Asset No. A069, Oticon Medical). Here, site preparation was performed at two specific feed rates (0.5 mm/s and 2.0 mm/s), and the temperature was measured using thermocouples as described above. Owing to limitations in the test rig, only the drilling procedures DP1 (standard with full irrigation), DP2 (reduced irrigation) and DP5 (no irrigation) were analysed. Ten drilling procedures were performed for each combination of drill system (MIPS, MONO), drilling protocol (DP1, DP2, DP5) and feed rate (0.5 mm/s, 2 mm/s), with each drill bit being exchanged for a new drill after being used five times.

The obtained data were imported and processed in Microsoft Excel (version 16.80). The change in temperature at each thermocouple position was calculated by subtracting the detected temperature from the experimental baseline temperature at the corresponding position. Since thermocouple measurements are sensitive to the distance between the heat source and the tip of the thermocouple, each osteotomy was scanned via computed tomography (Zeiss Metrotom 800 computed tomography, Zeiss Industrielle Messetechnik, Germany) and imported to Creo parametric version 7.0.2.0 (PTC Inc., Boston, USA), where the actual distance between each thermocouple channel and the drill tract could be determined for all positions. For each thermocouple measurement, a graph depicting the highest temperature in relation to the distance between the thermocouple tip and the final drill tract was generated. We established a power trend line equation and computed the error for each measurement in relation to this equation. The temperature data points were subsequently adjusted precisely to 0.5 mm, and the temperature was recalculated on the basis of the curve fit equation at this distance, considering the calculated error.

### Sampling and preparation of osteotomy and autologous bone chips

The effects of the drill system and protocol on the site were evaluated by drilling four fresh cadaveric cow tibia bones obtained from a local butcher. From these tibia samples, 48 cubical bone blocks approximately 10*10*8 mm in length were harvested by sawing (EXAKT® Apparatebau GmbH & Co, Norderstedt, Germany) (Fig 3B). The bone blocks were individually marked to randomize the subsequent site preparations across the individual bones and the extracted bone block location (proximal, central, or distal). Each block was subjected to a complete drilling procedure using either the MIPS or MONO system (Implantmed SI-923 Dental drill unit set at 2,000 rpm, Handpiece WI-75E/KM 20:1, W&H Nordic), generating an osteotomy. In accordance with the evaluation of heat generation described above, different protocols were used in order of assumed increased damage to the bone. Three different protocols were used, DP1 (full irrigation), DP4 (reduced irrigation and idling), and DP5 (no irrigation), for both the MONO and MIPS techniques, with eight drilling sequences for each combination of drill system and drilling procedure, resulting in a total of 48 osteotomies. After the drilling procedure was complete, the bone fragments generated during drilling were collected from the osteotomy area and surrounding area, as were the fragments stuck in the flutes of the drill bit. For each osteotomy, approximately half of the bone dust was transferred to a plastic vial filled with 4% paraformaldehyde for subsequent micro-CT and subsequent plastic embedding, while the other half was immersed in Hank’s balanced salt solution (HBSS, Life Technologies Limited, Paisley, UK) for subsequent analysis using Raman spectroscopy. The drilled blocks were longitudinally cut in half across the osteotomy and preserved in HBSS or 4% paraformaldehyde for subsequent Raman spectroscopy and histological assessments, respectively.

### Characterization of the osteotomy

#### Histology.

The bone half blocks (8 blocks for each combination of drill system and drilling protocol) with drilled cavities underwent a stepwise dehydration process in an ethanol series and were subsequently embedded in plastic resin (LR White, London Resin Co. Ltd, UK). A 50 µm thick central ground section was prepared from the embedded block and stained with toluidine blue, and the osteotomy roughness was qualitatively assessed using light optical microscopy (Nikon Eclipse E600; Nikon NIS-Elements software).

#### Raman spectroscopy.

Micro-Raman spectroscopy was performed using a confocal Raman microscope (Renishaw inVia Qontor) equipped with a 633 nm laser. The laser was focused down on to sample surface using a × 50 objective. The Raman scattered light was collected using a Peltier cooled CCD deep depletion NIR enhanced detector, behind a 2400 g mm^-1^ grating. The laser power at the sample was ~ 15 mW. Background subtraction and cosmic ray removal were performed using *intelligent spline* fitting in Renishaw WiRE 5.4 software. The composition of the bone in the vicinity of the drilled cavity was determined at four discrete points. The first two analyses (ID 1 and 3, in) were obtained in close proximity to the surface of the osteotomy, approximately 10–20 μm from the drill tract at least 2 mm apart from each other vertically. The remaining two points (ID 2 and 4, out) were positioned 490–500 μm from the first set, similar to the first group, which had a distance of at least 2 mm (Fig 3C). For each combination of drill system (MONO and MIPS) and drilling protocols (DP1, DP4 and DP5), eight samples were analysed. At each of the four discrete positions, spectra were acquired at 20 s integration time and 4 accumulations. The wavenumber axis was adjusted so that the ν_1_ PO_4_^3-^ peaks in all the spectra corresponded to ~959 cm^-1^. The baseline-corrected spectra were then normalized using Plot (http://plot.micw.eu/) to show equal intensities of the ν_1_ PO_4_^3-^ band in all the spectra. Curve fitting was performed using mixed Gaussian and Lorentzian functions, and the integral areas were quantified using MagicPlot (www.magicplot.com).

Compositional parameters such as mineral crystallinity, which is taken as the inverse full width at half-maximum (1/FWHM) of the ν_1_ PO_4_^3-^ peak [47]; the apatite-to-collagen ratio, also referred to as the mineral-to-matrix ratio (ν_2_ PO_4_^3-^/Amide III) [48]; and the carbonate–phosphate ratio (ν_1_ CO_3_^2-^/ν_1_ PO_4_^3-^) [49], were evaluated. The integral areas are as follows: ν_1_ PO_4_^3-^ (940–980 cm^-1^), Amide III (1210–1290 cm^-1^) and ν_1_ CO_3_^2-^ (1050–1080 cm^-1^).

### Characterization of the autologous bone fragments

#### X-ray micro-computed tomography (micro-CT).

The morphology of the autologous bone fragments dispersed in formalin was evaluated by microfocused X-ray computed tomography (microCT) using a Skyscan 1172 instrument (Bruker microCT, Kontlich, Belgium) operating at 70 kV with a 6.98 μm pixel size. Eight samples from each drill system (MONO and MIPS) and three different protocols (DP1, DP4, and DP5) were utilized for analysis. One hundred eighty-degree scans with 3-frame averaging at 0.5-degree rotation with n steps were employed. To reduce artefacts from low-energy X-rays, a 0.5 mm Al filter was applied. Reconstruction, visualization, and analyses were performed in the Skyscan software suite (NRecon, DataViewer, CTAn, CTVox, Bruker, Kontich, Belgium). The total volume of chips harvested from each osteotomy varied from sample preparation.

#### Backscattered electron scanning electron microscopy.

Four samples of bone chips were collected from each of the drill systems when used under the standard drilling protocol (DP1) and were embedded in resin (LR White Resin, London Resin Co. Ltd., UK), wet-polished with silicone carbide grinding paper (400–4000 grit) and air-dried before imaging. Low-vacuum backscattered electron scanning electron microscopy (BSE-SEM) was performed in a Quanta 200 environmental scanning electron microscope (FEI Company, The Netherlands) operating at 20 kV and 0.5 Torr water vapour pressure. Qualitative shape-related differences between the groups were evaluated at three different magnifications (100x, 200x, 400x) for each of the eight samples (four in each group).

#### Raman spectroscopy.

Raman spectroscopy was also employed to analyse the composition of the bone fragments. Eight samples from each drill system (MONO and MIPS) and three different protocols (DP1, DP4, and DP5) were utilized for analysis, with four acquisitions made at each position. Spectral acquisition and processing parameters were the same as those used for osteotomy evaluation, as described above. The samples were removed from Hank’s balanced salt solution (HBSS), placed on glass slides and left for approximately 10 minutes to allow the liquid to evaporate. Four spectra were subsequently acquired at 8 randomly allocated points for each of the 8 samples in each group. Like in the evaluation of the osteotomies, calculations were applied to determine the mineral crystallinity, the apatite-to-collagen ratio (ν_2_ PO_4_^3-^/Amide III), and the carbonate-to-phosphate ratio (ν_1_ CO_3_^2-^/ν_1_ PO_4_^3-^).

#### Statistical analysis.

The selection of ten drilling procedures for each combination was determined by the differences observed among groups in our initial data (showing a rise of 4.1 °C (SD 2.2, n = 15)). A power analysis indicated that eight drill passes would yield 80% statistical power with a 95% confidence level to detect a 3 °C increase in the average maximum temperature increase. The normal distribution of the data was verified via the Shapiro–Wilk test.

A two-way mixed ANOVA was run to evaluate the effects of the three types of drill bits as between-subject factors (MIPS GD, MIPS WD, MONO) on the drilling force and drilling torque with different feed rates (0.5, 1.0, 2.0 mm/s) as within-subject factors and including the interactions among factors. A similar analysis was conducted to evaluate the influence of the drill system (MIPS, MONO) and feed rate (0.5, 1.0, 2.0 mm/s) on the total mean work needed to generate the osteotomy.

The significance of the difference in heat generation between the subgroups of drill systems and drilling protocols was determined via two-way mixed ANOVA, with MIPS and MONO as between-subject factors and the drilling protocol (DP1, DP2, DP3, DP4, and DP5) as within-subject factors. Two analyses were performed: one when considering the position with the maximum temperature increase and one where the mean temperature increase across all probes was used. The influence of the feed rate on heat generation was evaluated via one-way repeated-measures ANOVA with a Bonferroni adjustment post hoc analysis for each drilling protocol and drill system.

For the analysis of the composition of drilled bone blocks and bone debris by Raman spectroscopy, one-way ANOVA and the Wilcoxon matched pairs signed rank test were used, where the groups on the surface of the osteotomy were paired to be further compared with those on the basis of distance. The bone fragment volume, surface area, thickness and structure model index (SMI) were compared via a parametric *t* test, with statistical significance evaluated at *p* < 0.05.

All the statistical tests were conducted using SPSS Statistics v.29.0.1.1 (IBM Corporation) and GraphPad Prism v.10.0.0 software (Boston, Massachusetts, USA), where *p* values *<* *0*.*05* were considered statistically significant. The mean values ± standard deviations are presented unless otherwise indicated.

## Results

### A parabolic drill has a greater cutting capability than a conventional twist drill

**Fig 4:**
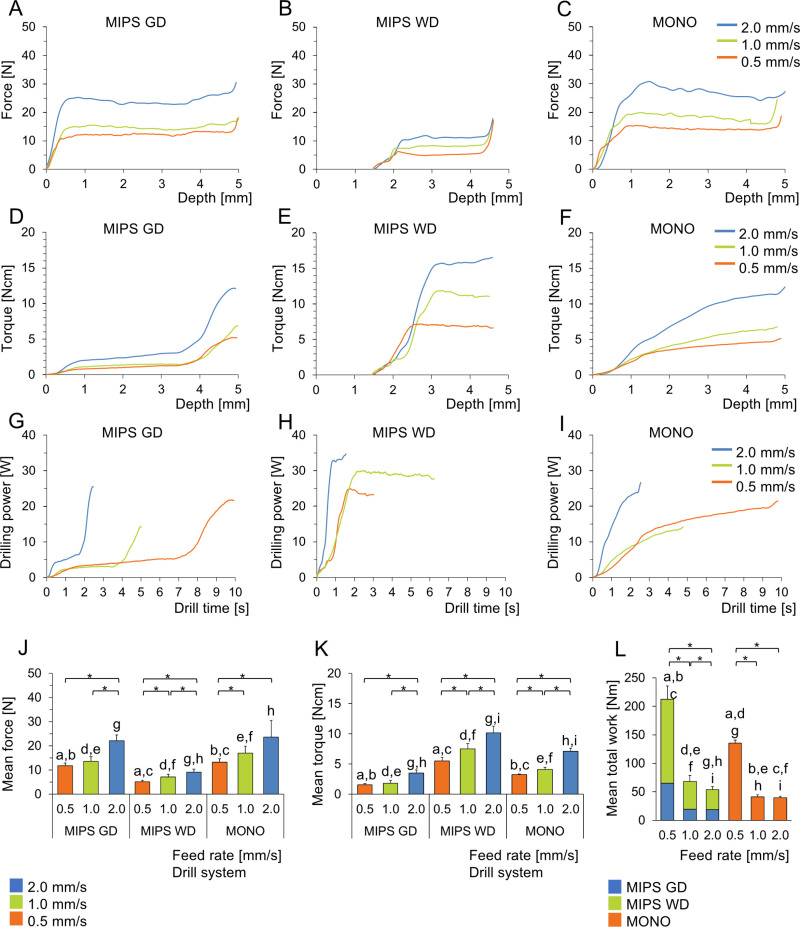
(A-C) Force versus drill depth and torque versus drill depth. (D-F) for the three drill bits. (G-I) Mean drilling power versus drilling time for the three drill bits. Mean thrust force (J) and mean torque (K) for the drill bits at feed rates of 0.5, 1.0 and 2.0 mm/s. The curves show the means from ten drilling procedures for each drill type. (L) Mean total work needed to generate the osteotomy for the drill systems MIPS and MONO at different feed rates. The data are presented as the means ± SDs. Bars that share the same letters are significantly different between the drill groups, whereas asterisks represent statistically significant differences within the drill group (p < 0.05; two-way mixed ANOVA). MIPS GD: MIPS Guide Drill, MIPS WD: MIPS Widening Drill.

Throughout the drill depth, variations in the drill design were reflected in the force curves, as indicated in Fig 4A-C. For all three drills, the force initially linearly increased before reaching a plateau. The torque measurements indicated a consistently low torque for the MIPS Guide drill until the larger diameter engaged with the bone, where the torque increased rapidly. The second drill step (Widening Drill) showed a steep increase in torque when engaging in the bone at 2 mm depth but reached a plateau at approximately 3 mm depth (Fig 4D-E). With the MONO drill, a more gradual increase throughout the osteotomy depth was observed (**Fig 4F**). Furthermore, both the force and torque increased with increasing feed rates, as shown in Fig 4A-F.

For all three drills and the tested feed rates, the mean force, torque, and total work were identified (Table 1). Statistically significant differences in the mean force and torque were observed between different feed rates for the MONO drill. Between 0.5 and 1.0 mm/sec in the MIPS GD, the torque and force differences were insignificant (Fig 4J,K). However, for MIPS WD, the mean force was significantly higher at 2 mm/sec. In general, the mean force required to feed the drill bit through the substrate was similar for the MIPS GD and MONO, whereas it was lower for the MIPS WD. This difference reflects the lesser amount of bone being removed during the widening of the osteotomy. In contrast, the torque was higher for the MIPS WD than for the other two drills.

**Table 1:** Mechanical data for the two drill bits in the MIPS system (MIPS GS, MIPS WD) and the single drill bits in the MONO system at different feed rates (mm/s). The data are shown as the means ± SD. MIPS GD: MIPS Guide Drill, MIPS WD: MIPS Widening Drill.

	Feed rate [mm/s]	MIPS GD	MIPS WD	MONO
Mean force [N]	0.5	11.66 ± 1.13	5.10 ± 0.63	13.25 ± 1.30
	1.0	13.57 ± 1.96	7.07 ± 1.17	16.91 ± 2.92
	2.0	22.05 ± 2.41	9.12 ± 1.23	23.54 ± 6.96
Mean peak force [N]	0.5	14.87 ± 1.11	17.18 ± 0.35	19.07 ± 3.00
	1.0	18.99 ± 1.36	12.95 ± 1.55	26.54 ± 7.14
	2.0	30.50 ± 3.19	17.88 ± 1.14	33.19 ± 8.97
Mean torque [Nmm]	0.5	15.47 ± 2.03	54.99 ± 5.91	32.54 ± 1.26
	1.0	17.83 ± 5.33	75.20 ± 8.80	41.01 ± 3.61
	2.0	35.02 ± 5.22	101.44 ± 11.20	70.94 ± 5.37
Mean peak torque [Nmm]	0.5	52.96 ± 9.83	77.34 ± 6.31	51.80 ± 1.39
	1.0	68.88 ± 14.24	122.64 ± 15.89	69.10 ± 5.80
	2.0	123.22 ± 17.34	173.47 ± 21.86	127.53 ± 9.80
Mean work [Nm]	0.5	64.76 ± 8.47	147.52 ± 15.80	135.28 ± 5.24
	1.0	21.75 ± 13.63	48.64 ± 5.68	41.33 ± 3.65
	2.0	19.10 ± 2.87	34.79 ± 3.84	39.27 ± 2.98

The drill power required to generate the osteotomy was affected primarily by the applied torque and the duration of the drilling sequence (Fig 4G-I). A higher feed rate resulted in a shorter drilling sequence, where feed rates of 0.5, 1.0 and 2.0 mm/sec corresponded to drill sequences of approximately 10, 5 and 2.5 s, respectively. The total amount of energy (or work) required to generate either the MIPS or MONO osteotomy, represented by the area under the curves (Fig 4G-I), decreased significantly with increasing feed rate, except for a nonsignificant difference between MONO at 1.0 and 2.0 mm/s (Fig 4L).

### A single-step drill system generates less heat than a three-step drill system

**Fig 5:**
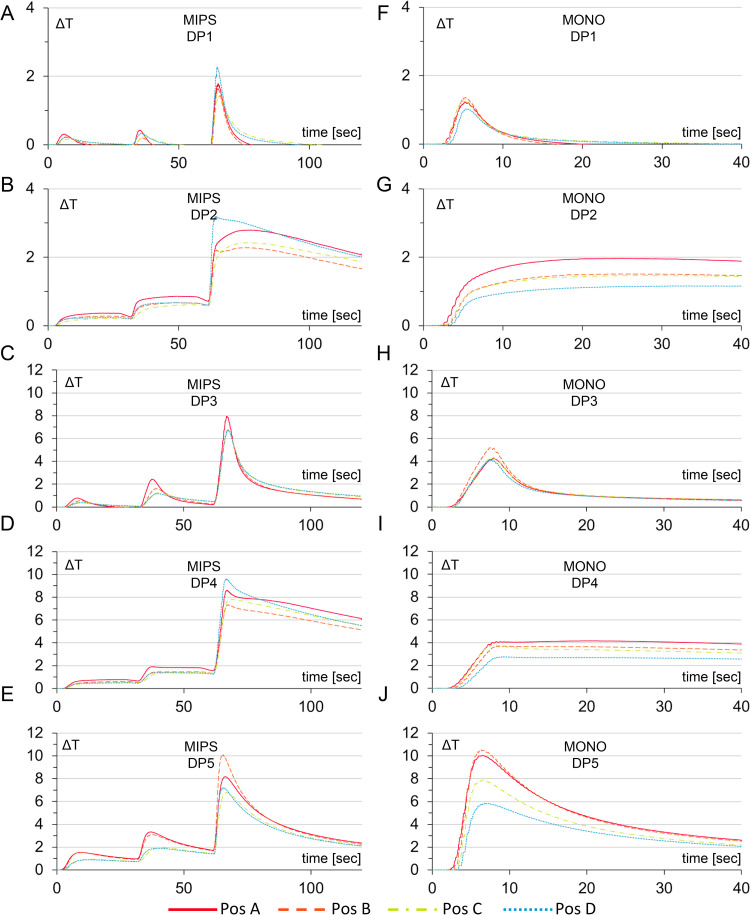
Graphs showing the temporal effects of temperature at the four different positions during a drilling sequence with guide drilling in two steps and final widening drilling (MIPS) and one-step drilling (MONO). For the first two drill steps, the temperature is lower since the thermocouples are more than 0.5 mm from the osteotomy. In the final step, all thermocouples are theoretically 0.5 mm from the osteotomy site. In these graphs, compensation for the actual thermocouple position has not been made. (A-E) Temperature increase for the MIPS system for the five different drilling protocols and (F-J) temperature increase for MONO for the five drilling protocols. DP1-standard drilling, DP2-reduced irrigation, DP3-idling, DP4-idling and reduced irrigation, and DP5-no irrigation.

The temporal temperature levels at each probe and for each combination of drill system and drilling protocol are illustrated in Fig 5. This includes guide drilling in two steps and final widening drilling for the MIPS and one-step drilling for the MONO systems. In the first two drill steps for the MIPS system, the temperature is lower since the thermocouples are more than 0.5 mm from the osteotomy site, whereas in the final step, all thermocouples are theoretically 0.5 mm from the osteotomy site. These graphs do not compensate for the actual thermocouple position, and the curve shapes reflect the presence or absence of irrigation in the protocol. The effect of reduced irrigation is evident in the curves (Fig 5H-I), with a higher and more drastic temperature increase at the beginning of the drilling. For drilling protocols with idling (DP2 and DP4) in MONO, the curves show a constant temperature increase, with both present and reduced irrigation (Fig 5B-G, D-I). In MIPS, these protocols also exhibit a temperature curve that does not drop throughout the sequence, unlike other protocols. Finally, for the no-irrigation protocol (DP5), both temperature curves for MIPS and MONO (Fig 5E-J) drastically increase and then decrease throughout the drilling.

**Fig 6:**
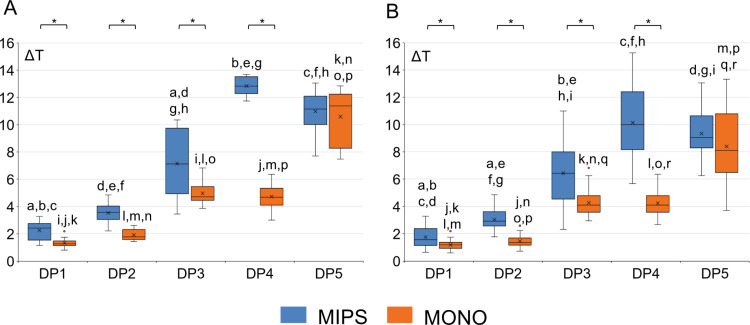
Graphs showing the mean maximum temperature increase at the position with the highest mean temperature increase (A) and the mean temperature increase across all probes (B) for each combination of drill system (MIPS and MONO) and drilling protocol (DP1-DP5). Asterisks indicate statistically significant differences between the MIPS and MONO systems within the same protocol. Letters a-r indicate statistically significant differences between different protocols within the same drill (two-way mixed ANOVA, p < 0.05). DP1-standard drilling, DP2-reduced irrigation, DP3-idling, DP4-idling with reduced irrigation, and DP5-no irrigation. ΔT is the temperature increase from the baseline in °C.

The mean maximum temperature increase at the probe position with the highest mean temperature increase and mean temperature increase across all probes for each combination of drill system (MIPS and MONO) and drilling protocol (DP1-DP5) was evaluated via two-way mixed ANOVA (Fig 6). The values of the highest mean temperature increase for different protocols are indicated in bold in Table 2.

**Table 2:** Increases in temperature for the two drill systems at different positions using different drilling procedures. The data in bold indicate the position where the highest mean temperature increase was registered. The data shown are the mean temperature increase ± SD in degrees centigrade. DP1-standard drilling, DP2-reduced irrigation, DP3-idling, DP4-idling with reduced irrigation, and DP5-no irrigation.

Drilling procedure	Drill system	Position				Mean
		A	B	C	D	
DP1	MIPS	1.79 ± 0.78	1.60 ± 0.76	1.36 ± 0.32	**2.26 ± 0.69**	1.75 ± 0.72
	MONO	1.26 ± 0.25	1.23 ± 0.29	**1.34 ± 0.38**	0.96 ± 0.22	1.20 ± 0.32
DP2	MIPS	**3.54 ± 0.69**	2.40 ± 0.44	3.06 ± 0.61	3.18 ± 0.52	3.05 ± 0.76
	MONO	**1.91 ± 0.44**	1.40 ± 0.33	1.43 ± 0.30	1.11 ± 0.25	1.46 ± 0.44
DP3	MIPS	**7.15 ± 2.39**	6.42 ± 2.07	6.85 ± 2.41	5.32 ± 1.84	6.44 ± 2.22
	MONO	4.11 ± 0.74	**5.00 ± 0.91**	4.18 ± 0.78	3.82 ± 0.62	4.27 ± 0.85
DP4	MIPS	10.53 ± 2.19	7.43 ± 1.66	**12.84 ± 0.67**	9.76 ± 2.39	10.14 ± 2.65
	MONO	4.44 ± 0.95	**4.73 ± 0.97**	4.24 ± 0.62	3.54 ± 0.47	4.24 ± 0.88
DP5	MIPS	9.83 ± 1.00	**10.98 ± 1.56**	8.58 ± 0.54	7.99 ± 1.03	9.35 ± 1.03
	MONO	9.60 ± 2.47	**10.59 ± 1.98**	7.38 ± 1.44	6.03 ± 1.65	8.45 ± 2.59

The statistical analysis revealed a statistically significant interaction effect between the drill system and the drilling protocol on the temperature increase, both when the probe with the maximum mean temperature increase and when the mean maximum increase across all the probes (*F*(2.487, 44.772) = 29.569, *p* < 0.001, partial η^2^ = 0.622 and *F*(2.743, 213.916) = 39.773, *p* < 0.001, partial η^2^ = 0.338, respectively) was considered. For both analyses and all drilling protocols, the temperature increase for the MONO system was statistically significantly lower than that for the MIPS system, except for the positive control (no irrigation), where a similar temperature increase was observed (Fig 6). Further analysis revealed a statistically significant effect of the drilling protocol on heat generation for both drill systems. In general, the mean maximum temperature increased with reduced irrigation and/or when the drilling time increased with idling. Notably, the MONO system was less sensitive to reduced irrigation, as demonstrated by the lack of a significant difference between the drilling protocol where idling (DP3) was applied and the drilling protocol in which the combined effect of idling and reduced irrigation (DP4) was evaluated. Furthermore, the standard deviation for the MIPS system was generally greater than that for the MONO system.

**Fig 7:**
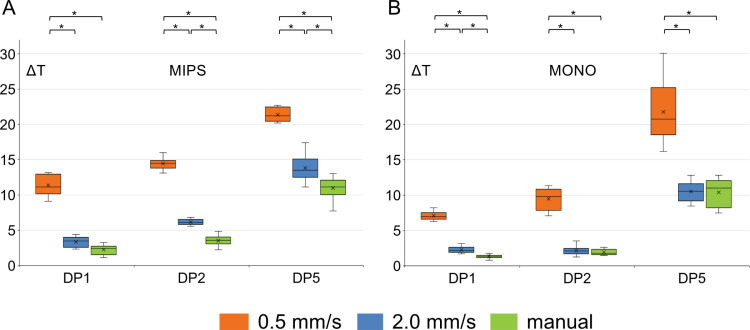
Graphs showing the mean temperature increase at the positions with the highest mean temperature increase for the MIPS (A) and MONO (B) systems and three drilling protocols (DP1, DP2, and DP5) when drilling at feed rates of 0.5 and 2.0 mm/s and using a manual drilling procedure. DP1-standard drilling, DP2-reduced irrigation and DP5-no irrigation. *Statistically significant difference in temperature increase between the three different feed rates within the same protocol (one-way repeated-measures ANOVA, p < 0.001). ΔT is the temperature increase from the baseline in °C.

Two different feed rates (0.5 mm/s and 2.0 mm/s) were evaluated for three drilling protocols (DP1, DP2, and DP5) and compared with the data obtained when drilling using a manual procedure (Fig 7). The values of the highest mean temperature increase for different protocols and feed rates are indicated in bold in Table 3. An increase in the feed rate from 0.5 mm/s to 2 mm/s elicited statistically significant changes in heat generation for both drill systems. For example, for the MIPS system with full irrigation (the standard protocol, DP1), increasing the feed rate from 0.5 to 2 mm/s resulted in a decrease in heat generation from 11.36 ± 1.48 to 3.35 ± 0.70 °C when considering the probes with the highest mean temperature increase, with a statistically significant decrease of 8.07 (95% CI, -9.65 to -6.39) °C, *p* < 0.0001. Similarly, for the MONO system under the same conditions, heat generation decreased from 7.08 ± 0.56 to 2.26 ± 0.47 °C, yielding a statistically significant decrease of 4.83 (95% CI, -5.37 to -4.28) °C, *p* < 0.0001. Similar reductions were observed for the drilling protocol with reduced irrigation (DP2), where heat generation decreased from 14.45 ± 0.78 to 6.16 ± 0.40 °C (8.29 (95% CI, -9.07 to -7.51) °C, *p* < 0.0001) and 9.48 ± 1.56 to 6.16 ± 0.40 °C (7.32 (95% CI, -8.92 to -5.73) °C, *p* < 0.0001) for the MIPS and MONO systems, respectively. The temperature increase during manual drilling was either similar to or lower than that during drilling at 2.0 mm/s, indicating that manual drilling was performed at a feed rate at or slightly above 2.0 mm/s (Fig 7).

**Table 3:** Increases in temperature for the two drill systems at different positions using different drilling procedures and feed rates. The data in bold indicate the position where the highest mean temperature increase was registered. The data shown are the mean temperature increase ± SD in degrees centigrade. DP1-standard drilling, DP2-reduced irrigation, DP3-idling, DP4-idling with reduced irrigation, and DP5-no irrigation.

Drilling procedure	Drill system	Feed rate [mm/s]	Position				Mean
			A	B	C	D	
DP1	MIPS	0.5	11.28 ± 2.28	**11.36 ± 1.48**	7.56 ± 1.56	10.10 ± 1.70	10.08 ± 2.31
		2.0	**3.35 ± 0.70**	2.78 ± 0.33	2.54 ± 0.67	2.27 ± 0.91	2.73 ± 0.97
	MONO	0.5	5.61 ± 0.84	5.24 ± 0.79	5.24 ± 1.76	**7.08 ± 0.56**	5.79 ± 1.31
		2.0	1.99 ± 0.35	**2.26 ± 0.47**	1.56 ± 0.27	1.61 ± 0.90	1.85 ± 0.61
DP2	MIPS	0.5	**14.45 ± 0.78**	11.32 ± 1.10	8.49 ± 2.24	10.34 ± 1.41	11.15 ± 2.61
		2.0	5.81 ± 0.73	**6.16 ± 0.40**	4.38 ± 0.74	5.95 ± 0.81	5.57 ± 0.97
	MONO	0.5	8.48 ± 1.46	**9.48 ± 1.56**	6.45 ± 0.97	6.25 ± 1.68	7.66 ± 1.95
		2.0	2.00 ± 0.48	**2.15 ± 0.63**	2.12 ± 0.54	2.01 ± 0.56	2.08 ± 0.54
DP5	MIPS	0.5	19.40 ± 2.68	**21.39 ± 0.94**	14.44 ± 2.26	19.92 ± 3.62	18.79 ± 3.62
		2.0	12.54 ± 2.33	13.26 ± 2.03	11.96 ± 1.68	**13.83 ± 1.86**	12.90 ± 2.04
	MONO	0.5	17.38 ± 1.65	**21.80 ± 4.38**	16.40 ± 0.84	12.09 ± 3.54	16.80 ± 4.49
		2.0	9.51 ± 1.89	**10.52 ± 1.47**	7.69 ± 1.78	6.68 ± 1.31	8.60 ± 2.18

### The absence of irrigation causes changes in bone composition at the osteotomy site

**Fig 8:**
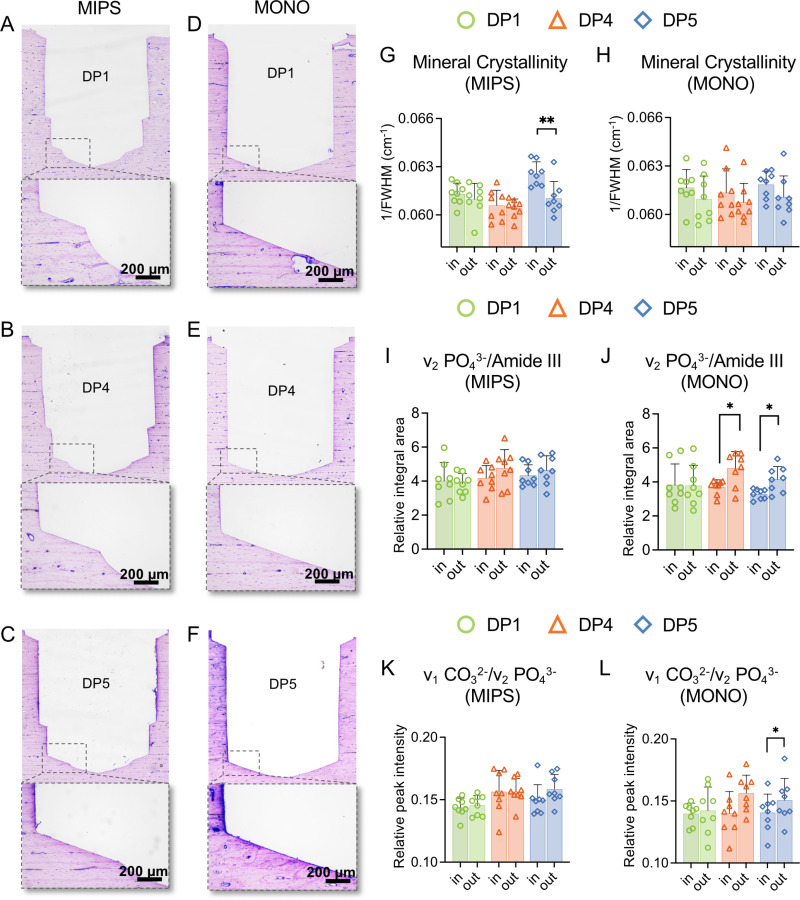
Histologic evaluation of the osteotomy site. Undecalcified toluidine blue-stained sections of the drilled osteotomies for MIPS (A-C) and MONO (D-F) with standard drilling (DP1), idling with reduced irrigation (DP4), and no irrigation (DP5) protocols. (G-L) Raman data evaluated at 10–20 μm from the osteotomy site (in) and 490–500 μm away from it (out), where (G-H) represents crystallinity, (I-J) is the carbonate–to–phosphate ratio (ν_1_ CO_3_^2-^/ν_1_ PO_4_^3-^), and (K-L) is the PO_4_^3-^/Amide III ratio. (p < 0.05; one-way ANOVA). DP1-standard drilling, DP4-idling with reduced irrigation, DP5-no irrigation.

For both the MIPS and MONO systems, smooth and intact osteotomy cuts were generally observed. However, among the performed protocols, the surface of the osteotomy of nonirrigated drilling (DP5) for both MIPS and MONO appeared to be rougher (Fig 8C, F), where the roughness of the surface increased with increasing bluish color, indicating more microcracks and debris within the osteotomy.

From the bone composition evaluation, changes were most evident in the groups with idling and reduced irrigation (DP4) and nonirrigated drilling (DP5). In the MIPS system, mineral crystallinity was significantly greater at the surface of the osteotomy site than farther away for the no-irrigation procedure (p = 0.0078). To investigate further, the apatite-to-collagen and apatite-to-collagen ratios were calculated, but no significant correlations were found for the MIPS groups. However, for MONO, in DP4 and DP5, the apatite-to-collagen ratio was greater farther from the osteotomy, and for DP5, the carbonate-to-phosphate ratio also increased at distant positions (Fig 8J,L). The Raman spectra for the groups in which any significant changes were observed are also displayed in S2 Fig. Overall, while statistical analysis revealed significant differences in composition for certain protocols, pairing was found to be significant only for the ν_1_ CO_3_^2-^/ν_1_ PO4^3-^ ratio in DP5 and MONO between the chosen groups (ID 1 and 3, in) and (ID 2 and 4, out).

### Characterization of bone fragments

**Fig 9:**
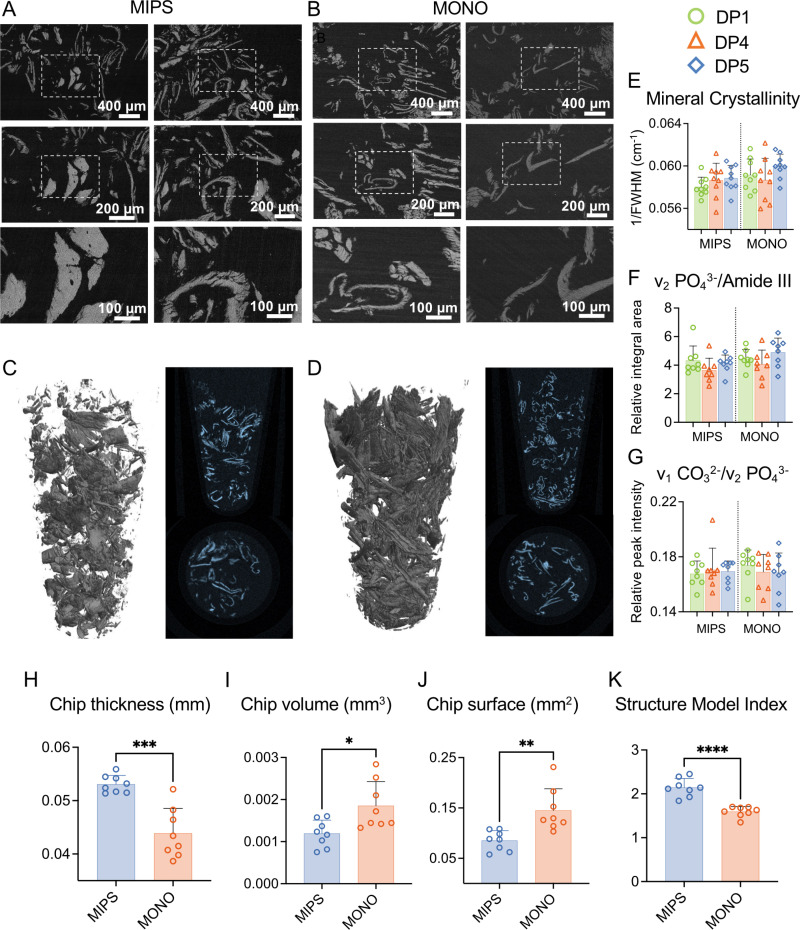
Images and quantitative results from bone fragment evaluation. (A-B) SEM images of bone chips from (A) MIPS and (B) MONO standard drilling (DP1) at 100x, 200x and 400x magnification. (C-D) Micro-CT 3D reconstructions of bone fragments. (E-G) Raman data of bone chips obtained from MIPS and MONO by three drilling protocols (DP1, DP4 and DP5), where (E) represents crystallinity, (F) is the carbonate-to-phosphate ratio (ν_1_ CO_3_^2-^/ν_1_ PO_4_^3-^) and (G) is the ν_2_ PO_4_^3-^/Amide III ratio (p < 0.05; one-way ANOVA). Comparisons of (H) chip thickness, (I) chip volume, (J) chip surface area and (K) structure model index obtained from microCT data quantification (Student’s *t t*est, *p* < 0.05). DP1-standard drilling, DP4-idling with reduced irrigation, DP5-no irrigation.

According to the results (Fig 9C-D and H-K), MONO results in large but thinner shavings of bone, whereas MIPS results in a less homogenous population of particles. 3D analysis revealed that the volume and surface area of the chips generated from the MONO drill were greater than those generated from the Guide and Wide Drills (p > 0.05). The structural model index was lower for the MONO drill-generated fragments than for the MIPS drill-generated fragments, confirming that the former had a more plate-shaped morphology. Although, for both drilling techniques, the shape of the bone chips varied greatly, the qualitative SEM image analysis results were consistent with those obtained from structural analysis. The MIPS drill generated thicker chips, whereas the MONO drill produced relatively less thick bone fragments (Fig 9A-B). Also, the evaluation of bone chips morphology for non-standard protocols did not reveal any additional correlations (S3 Fig). To further evaluate the composition of the bone chips, Raman data were investigated; however, no differences were identified between the groups (Fig 9E-G).

## Discussion

The thermal and mechanical effects of bone drilling are critical aspects to consider in various medical procedures, especially when it involves the subsequent need for osseointegration. The present results demonstrate that osteotomy for a bone-anchored hearing implant can be created using a one-step drilling protocol with the MONO drill system while keeping the force and torque within clinically acceptable limits. This is a demonstration of the cutting efficiency of the MONO drill design since the volume of bone removed using the MONO drill is 60% larger than the volume removed in the first two steps via the guide drill in the MIPS systems. Compared with the three-step drill system, the superior cutting performance of the MONO drill design leads to less total energy being required for osteotomy creation. This energy is then translated to heat and distributed to the surrounding bone, the bone dust, the drill bit and the irrigation fluid. Interestingly, the temperature increase in the substrate surrounding the osteotomy was significantly lower for the one-step drill system than for the conventional three-step approach, which also deviated from the recommended procedure in terms of the amount of irrigation and idling. Furthermore, the drilling duration significantly influenced the energy needed for osteotomy creation, which was also reflected in the reduced temperature increase in the substrate when the feed rate was increased. Taken together, these results suggest that the MONO drill system is less affected by alterations in the drilling protocol, such as reduced irrigation, idling and drilling duration, hence making it more forgiving to inexperienced surgeons.

## Mechanical characteristics of the drill system

A risk associated with increased amounts of bone removal and increased feed rates is clogging of the chips, leading to rapid increases in force and torque [50]. For all three drill bits, a significant increase in both the maximum force and torque was observed with increasing feed rate. Potentially, clogging could be a risk for the MONO system since all the bone is removed in one single drill step; however, the increase in force and torque was gradual, and no indication of clogging was observed at the highest feed rate of 2 mm/s. This was potentially avoided because of the increased space available for bone chip evacuation, and moreover, the depth of the osteotomy was only 4.75 mm. The hypothesis regarding the increased performance of the MONO drill suggests that its design, with less metal in contact with the osteotomy surface during drilling, results in reduced friction and consequently less energy required for bone removal [51]. High friction can harm the drill bit and reduce its cutting capacity [10]. This notion is supported by the more gradual increase in torque throughout the osteotomy creation, in contrast to the sharp increase observed for the two MIPS drills. Interestingly, the total amount of work needed to generate the osteotomy decreased with increasing feed rate, particularly when the effective drilling time decreased from approximately 9.5 s (0.5 mm/s) to 4.8 s (1 mm/s), whereas the decrease was less pronounced when the drilling time decreased to 2.4 s (2 mm/s).

### Influence of drill design on heat generation

During the cutting process, bone chips are created at the junction between the cutting edge of the drill bit and the substrate. The heat generated during this process is distributed to the drill bit itself, to the surrounding substrate, to the cooling fluid and to the chips. Studies of metal machining have shown that most of the heat created dissipates to the bone chips and, to a lesser extent, to the drill bit and substrate [52,53]. Hence, efficient removal of hot chips is essential for maintaining heat generation at an acceptable level. Surprisingly, despite removing all the bone in one step and not three steps, the MONO system generated less heat within the bone compared with the MIPS system. In contrast, an *in vitro* study using a guided osteotomy technique reported a lower maximum temperature increase, determined using infrared thermography, for sequential drilling with four drills to generate an osteotomy of 4.2 mm in diameter than for a one-step drilling sequence, whereas the opposite was found for the conventional approach without a guide [54]. In this investigation, drills with a conventional twist drill design were used. Similarly, Möhlhenrich et al reported a significantly greater temperature increase for a single drill osteotomy with a final diameter of 4.2 mm than for a three-step approach in polyurethane blocks with a density of 0.48 g/cm^3^ [55]. Additionally, comparisons were made using conventional dental twist drills of different diameters. In contrast, Koutiech et al investigated the maximum heat generation during site preparation for a 4.25 mm dental implant by comparing single- and five-step drilling protocols. In this study, the single drilling protocol generated less heat than the conventional method did [56]. Importantly, while the gradual drilling protocol used conventional dental twist drills, the drill used in the single protocol had three straight flutes and wider channels. The authors therefore attributed the increased performance to improved elimination of bone chips and a reduced drill–bone contact area, leading to lower frictional heat. Cseke and Heinemann investigated the effects of drill speed and feed rate on heat generation in artificial bone blocks and cadaveric bone specimens and suggested that the main heat source does not originate at the cutting point but rather involves friction between the bone chips in the drill flutes and the borehole wall [57]. Similarly, the likely reason for the enhanced performance of the MONO drill is the increased space available in the flute of the MONO drill bit compared with the more traditionally designed twist dills in the MIPS system. This would result in more space available for the hot bone chips to be removed and transported from the osteotomy and a better exchange of cooling fluid. The MIPS drill system features a conical working section (in the MIPS Guide Drill), which increases the overall contact area during operation, including both the cutting edge and the sidewall. This larger contact area may result in greater frictional forces and, consequently, higher heat generation. In contrast, the MONO system utilizes a straight drill design, where pressure transmission is primarily confined to the cutting edge during operation, leading to reduced contact with the osteotomy sidewall and less friction. Furthermore, the slimmer design of the MONO drill leads to less metal being in contact with the bone, potentially contributing to reduced friction and thereby less heat buildup.

The MIPS system was previously evaluated using the same method used in the present study to investigate heat generation at different drilling procedures (DP1-DP5) [24]. The temperature increase in the standard protocol with full irrigation (DP1) was greater (5.5 ± 0.8 °C) than that in the present evaluation (1.34 ± 0.38 °C). The previous evaluation also revealed a high sensitivity to reduced irrigation, with mean maximum temperature increases of 16.3 ± 3.9 °C and 17.8 ± 1.8 °C in the cases of reduced irrigation (DP2) and idling with reduced irrigation (DP4), respectively. The corresponding heat increases for MISP in the present evaluation were 3.5 ± 0.7 and 10.5 ± 2.2 °C for DP2 and DP4, respectively. The likely reason for these differences in heat generation is the design change of the MIPS drills, which were introduced following a multicenter, randomized, controlled, clinical study reporting a nonsignificantly higher implant extrusion rate for the MIPS group than for the group installed using the traditional linear incision technique [23,58]. The modified MIPS drills, which were also evaluated here, were subsequently evaluated in an exploratory pilot study, which reported a trend toward a lower implant loss rate for the modified system than for the original system [32]. The authors reasoned that the greater degree of implant loss in the original MIPS group may be a result of impaired osseointegration caused by overheated bone.

### Influence of the feed rate on heat generation

Drilling energy is defined as the energy needed for producing a hole and implies that a higher rate of energy consumption would lead to greater heat generation during the drilling sequence. Hence, the commonly used parameters when studying drilling processes for machining have been less commonly used in studies of bone drilling and reported in the medical literature [24]. A lower cutting energy is associated with less residual and thermal damage in the cutting region, whereas a higher energy consumption implies greater heat generation, leading to a greater rise in temperature [59].

The heat generation in the bone decreased with increasing feed rate in both evaluated drill systems. This finding aligns with the mechanical observations where less energy was required when drilling at a higher feed rate and agrees with other studies where the influence of the feed rate on heat generation was studied [60]. An evaluation of porcine femoral diaphysis revealed that an increase in the feed rate caused a significantly lower increase in the bone temperature, as determined by measurements with a thermocouple 0.5 mm from the drill site [61]. For a given spindle speed, the shearing energy required to cut the bone material also increases with increasing feed rate; a large portion of this energy is converted into heat. On the other hand, the drilling process is completed in a much shorter time at higher feed rates. The shorter drilling time not only results in a shorter time of exposure to heat generation but also reduces the heat transferred to the bone from the hot drill bit [19].

### Influence of the drilling protocol on heat generation

The amount of irrigation influences heat generation during bone drilling. For a guided drill system, there are potential drawbacks with the guide, including less efficient penetration of the cooling fluid and added heat generation due to the friction between the drill and the guide [62–64]. Both the MIPS and MONO systems generated increased heat generation when they deviated from the standard protocol. However, the single drill system was less sensitive than the three-step drill system was, as indicated by the lower mean maximum temperature increase. The drilling for preparation of the osteotomy for a bone-anchored hearing implant is performed manually, and many parameters, such as the force applied, feed rate (level, constant, intermittent), angle of the drill bit, amount of irrigation provided and extent of idling once the drill reaches its final depth, are controlled by the surgeon and assistant. Therefore, a more robust system that is less sensitive to deviations is beneficial.

### Bone composition in the osteotomy

The exact threshold for heat-induced trauma to the bone has not been clearly defined, although it has become a “dogma” that the safe range is between 47–55 °C for a drilling sequence lasting > 1 minute [65]. Temperatures exceeding 50 °C have been demonstrated to cause irreversible alterations in bone structure and physical properties [66,67]. Necrotic bone is broken down by osteoclast activity, which can jeopardize the stability of bone screws and pins, potentially compromising the healing process, affecting bone viability and leading to complications such as delayed healing of defects and thermal osteonecrosis [28,38,68–70]. Several studies have explored the effects of drilling parameters in vitro, identifying external irrigation as one of the main factors influencing the increase in temperature [71]. Although temperatures over 50 °C are associated with irreversible changes in bone structure and physical properties, in some works, a temperature increase in bone above the “safe” temperature range does not induce any bone necrosis [72]. However, when a new drill system is introduced into clinics, the dogma remains the same. Additionally, it should be noted that in this study, the temperatures were measured 0.5 mm away from the osteotomy site, so they do not reflect the exact temperature to which the bone at the osteotomy site was exposed.

In the present study, in the MIPS and MONO systems, the differences in bone composition within the osteotomy were primarily identified in the no-irrigation and reduced-irrigation protocols, which presented the greatest temperature increases. This finding is consistent with previously conducted studies showing that the primary factor controlling heat generation during bone drilling is external irrigation [71], although both the drilling parameters and the drill itself significantly contribute to changes in bone composition [42]. In the MIPS system (DP5), greater mineral crystallinity was found at the osteotomy site. However, it should be mentioned that during measurements, signal acquisition was challenging at the osteotomy surface because of noise from bone dust stuck in the osteotomy walls. While statistical analysis revealed significant differences in composition for certain protocols, pairwise comparisons indicated effective pairing in only one group. In the MONO system, the organic component ratio was greater closer to the osteotomy site. This suggests that thermal and mechanical effects may have removed the phosphate surrounding the collagen while leaving the collagen intact because of its greater interconnectedness. For further analysis, histomorphometry could be performed to quantify empty lacunae and lacunae with osteocytes on the osteotomy surface, as was done by Alam et al. [10].

### Composition of the bone chips

Excessive heat not only potentially results in thermal necrosis, causing damage to the bone and surrounding structures but also influences the status and shape of the bone particles remaining in the osteotomy after insertion [41]. Therefore, bone fragments were investigated for their morphological and compositional differences, as they may also play an important role in implant osseointegration. The instructions for use of the MIPS and MONO systems stipulate that the osteotomy should be flushed after each drill step, thereby removing the remaining bone chips from the osteotomy site. [73]. However, it remains unclear whether the bone fragment generated during drilling should remain within the osteotomy space or be removed as conventionally done. Research has suggested that leaving bone fragments within the osteotomy space might have an osteogenic effect, enhancing bone healing and subsequent implant osseointegration [41]. A rat study demonstrated that leaving bone fragments within the osteotomy not only does not impair implant osseointegration but also, if the bone debris are viable, can lead to earlier peri-implant bone formation [74,75]. In this study, we observed that chips created by the MONO procedure are more homogeneous in size, which can be explained by the fact that there is just one drilling step, thereby reducing variational changes between different samples. Despite the morphological differences in bone fragments between the two groups, no compositional changes were detected. Further in-depth studies are necessary to determine the potential effects of these bone fragments on implant osseointegration.

### Methodological considerations

To avoid consuming donated human temporal bone samples, the mechanical properties of the drill bits were investigated using fresh cow tibias. The cortical layer in the bovine tibia is more than 7 mm thick [76], and even though the cow tibia cortex does not fully replicate human temporal bone, it is considered to have comparable properties, allowing for controlled in vitro drilling tests [77,78]. In a previous study, we obtained mechanical material when drilling artificial bone blocks (Sawbones, PCF 50) [24]; however, studies have shown that this material is not comparable to bone when force and torque are considered [57]. Here, the polyurethane foam blocks were used for thermal effects analysis. While polyurethane foam does not fully replicate the properties of living human temporal bone, it offers consistent characteristics to assess temperature elevation during drilling [79]. Another crucial benefit is that it facilitates precise and standardised placement of thermocouples and subsequent scanning, tasks that are significantly more challenging in bone.

Mechanical characteristics and heat generation were only evaluated at a drill speed of 2,000 rpm since this is the traditionally used drill speed for BAHSs. However, it is evident from the literature that the spindle speed influences both parameters, with a general conclusion that an increase in the spindle speed increases the bone temperature, at least for low-speed drilling (up to 3,000 rpm) [43,80]. To address the risk of inconsistencies due to the lack of control over the irrigation flow rate, the procedure was manually performed by the same operator using a 20 ml syringe. For the determination of the temperature increase during drilling in bone or artificial bone, two methods have been employed: thermocouples and infrared thermography. Both are associated with their own set of drawbacks, where the thermocouple is limited to measuring the temperature increase at a single point and is sensitive to the distance to the heat source, whereas infrared thermography can detect only the surface temperature [34,43,54]. Here, we were interested in recording the temperature increase within the bone rather than the increase in the bone chips, cooling fluid and drill bit. Moreover, a comparison with a clinically used system is made; hence, the relative difference is of interest. To address the drawbacks of using thermocouples, a strategic approach was adopted: four thermocouples were positioned along the osteotomy, and after completion of the drilling, compensation for possible discrepancies in the distance to the heat source was performed. Importantly, multiple parameters interact and contribute to the heat profile when drilling in bone, including bone quality and cortical bone thickness; the irrigation method; the temperature of the irrigation fluid; the feed rate; the rotational speed; the drill depth and diameter; and design aspects such as the rake angle, tip angle, and flute design, as well as the experimental setup and methods used to capture the temperature [8,43,80,81]. Therefore, comparisons between experimental studies are difficult since multiple parameters differ. In the present experiments, a comparison between a clinically successful system (MIPS) and a new system is performed, and hence, the difference in performance and outcome between them are of perhaps more interest than the absolute values and their relation to previous studies.

### Clinical implications

A single-drill protocol reduces the risk of drill trajectory misalignment compared to a multidrill approach, which is particularly important during flapless procedures where visibility is limited [82,83]. Misaligned drilling can lead to a suboptimal osteotomy shape, improper implant alignment, and delayed healing, ultimately jeopardizing the implant’s primary stability and subsequent osseointegration [4–6].

The MONO single-drill system for BAHS was introduced in clinical practice in 2021. A retrospective study of 18 bone-anchored hearing implants installed with MONO demonstrated reduced surgical complexity, a shortened surgery time, a low degree of intra- and postoperative complications, and no implant losses, although the men’s follow-up time was just over one month [84]. More recently, a prospective, multicentre study investigated the clinical outcomes of bone-anchored hearing implant surgery using the MONO procedure in 51 adult patients across seven centres [85]. No severe intraoperative complications were reported, with surgery lasting 10 minutes on average, and at three months, 94.2% of the implant/abutment complexes provided reliable anchorage for sound processor usage. Four implants were lost due to trauma in two patients: one implant was extruded due to incomplete insertion, and one implant was extruded spontaneously.

These findings suggest that the MONO procedure offers a safe and efficient surgical technique for inserting bone-anchored hearing implants, with the potential for further improvement in patient outcomes. By utilizing a one-step drilling sequence, both the drilling and the length of the implantation procedure is shortened, thereby reducing the discomfort for the patient. Additionally, fewer intra-operative complications may be expected due to the simplified drilling procedure. However, long-term follow-up studies are necessary to better assess the safety and effectiveness of the MONO system.

## Conclusion

The results of this study demonstrate that heat generation in the bone surrounding an osteotomy during the drilling procedure is influenced by the number of drilling steps, drill design, feed rate, and amount of irrigation. Notably, the single drill system generated significantly less heat than the with a three-step system did, which was attributed to the design of the single drill system, with a slim macro design leading to increased space in the flutes for evacuation of the bone chips. This also highlights the importance of considering multiple aspects when designing and evaluating a drill system. Moreover, the single drill system was less sensitive to variations in drilling sequence execution as well as the amount of irrigation. This will potentially increase the likelihood of success when introduced in clinical practice providing a safe and efficient system for osteotomy creation for bone-anchored hearing implants.

## Supporting information

S1 Fig Drilling test machine. The following drill machine is used for determining the insertion torque in artificial bone for the implants in the percutaneous bone-anchored system. The insertion torque is measured at constant feed rate using a specially designed rig as presented in the figure. There, (A) represents the overview of the apparatus, (B) shows the fixation mechanism for the polyurethane blocks, (C-D) present an example of drilling with MONO drill through the cannula.
(PDF)

S2 Fig Micro-Raman spectroscopy of bone chips. (A-C) Raman curves of drilling protocols where significant differences were identified. (A) MIPS and (B) MONO were performed by idling with reduced irrigation (DP4), and MONO (C) was conducted with no irrigation protocol (DP5). Here, (1–4) represent the spectra obtained with the proximity of 10–20 µm to the osteotomy (1,3) and 450–500 µm farther away (2.4).
(PDF)

S3 Fig Morphology of bone chips. Comparisons of the chip thickness, chip volume, chip surface area and structural model index obtained from microCT data quantification for (A-D) DP4, (E-H) DP5 and (I-L) DP + DP4 + DP5 combined (parametric *t* test, *p* < 0.05). There, DP1-standard drilling, DP4-idling with reduced irrigation, and DP5-no irrigation.
(PDF)
