# Supplementary material for: Mechanical and thermal efficiency of a single drill system for bone-anchored hearing implants
Source: PLoS One. 2025 May 30;20(5):e0311026. doi: 10.1371/journal.pone.0311026 (PMC12124499; doi:10.1371/journal.pone.0311026)
Supplement: S1 Fig — The following drill machine is used for determining the insertion torque in artificial bone for the implants in the percutaneous bone-anchored system. The insertion torque is measured at constant feed rate using a specially designed rig as presented in the figure. There, (A) represents the overview of the apparatus, (B) shows the fixation mechanism for the polyurethane blocks, (C-D) present an example of drilling with MONO drill through the cannula. (PDF) [file pone.0311026.s001.pdf]

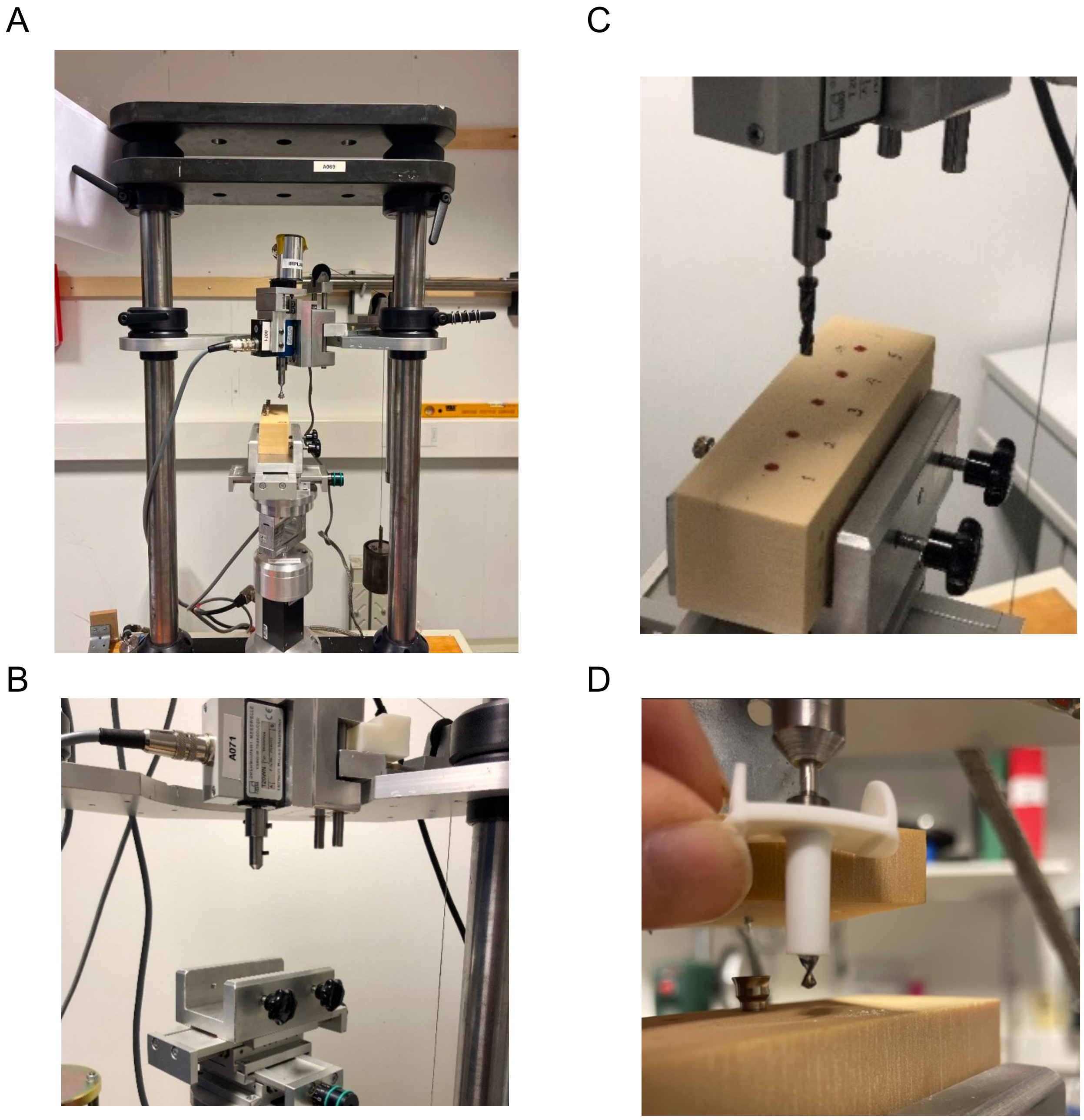

S1 Fig. Drilling test machine. The following drill machine is used for determining the insertion torque in artificial bone for the implants in the percutaneous bone-anchored system. The insertion torque is measured at constant feed rate using a specially designed rig as presented in the figure. There, (A) represents the overview of the apparatus, (B) shows the the fixation mechanism for the polyurethane blocks, (C-D) present an example of drilling with MONO drill through the cannula.
