# Supplementary material for: Mechanical and thermal efficiency of a single drill system for bone-anchored hearing implants
Source: PLoS One. 2025 May 30;20(5):e0311026. doi: 10.1371/journal.pone.0311026 (PMC12124499; doi:10.1371/journal.pone.0311026)
Supplement: S2 Fig — (A-C) Raman curves of drilling protocols where significant differences were identified. (A) MIPS and (B) MONO were performed by idling with reduced irrigation (DP4), and MONO (C) was conducted with no irrigation protocol (DP5). Here, (1–4) represent the spectra obtained with the proximity of 10–20 µm to the osteotomy (1,3) and 450–500 µm farther away (2.4). (PDF) [file pone.0311026.s002.pdf]

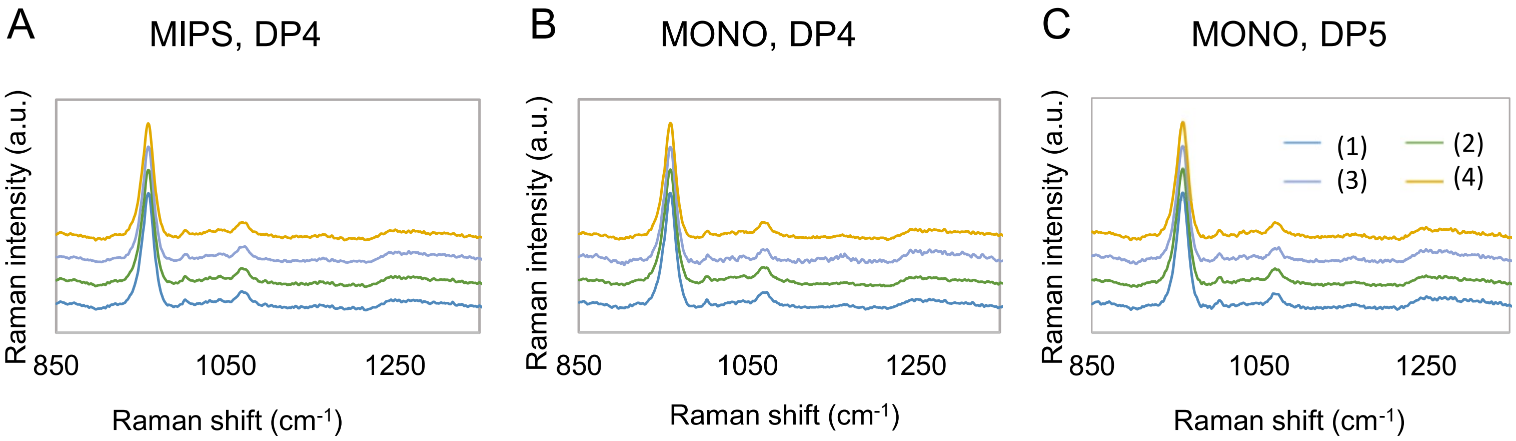


**S2 Fig. Micro-Raman spectroscopy of bone chips**. (A-C) Raman curves of drilling protocols where significant differences were identified. (A) MIPS and (B) MONO were performed by idling with reduced irrigation (DP4), and MONO (C) was conducted with no irrigation protocol (DP5). Here, (1-4) represent the spectra obtained with the proximity of 10-20 μm to the osteotomy (1,3) and 450-500 μm farther away (2.4)
