# Supplementary material for: Mechanical and thermal efficiency of a single drill system for bone-anchored hearing implants
Source: PLoS One. 2025 May 30;20(5):e0311026. doi: 10.1371/journal.pone.0311026 (PMC12124499; doi:10.1371/journal.pone.0311026)
Supplement: S3 Fig — Comparisons of the chip thickness, chip volume, chip surface area and structural model index obtained from microCT data quantification for (A-D) DP4, (E-H) DP5 and (I-L) DP + DP4 + DP5 combined (parametric t test, p < 0.05). There, DP1-standard drilling, DP4-idling with reduced irrigation, and DP5-no irrigation. (PDF) [file pone.0311026.s003.pdf]

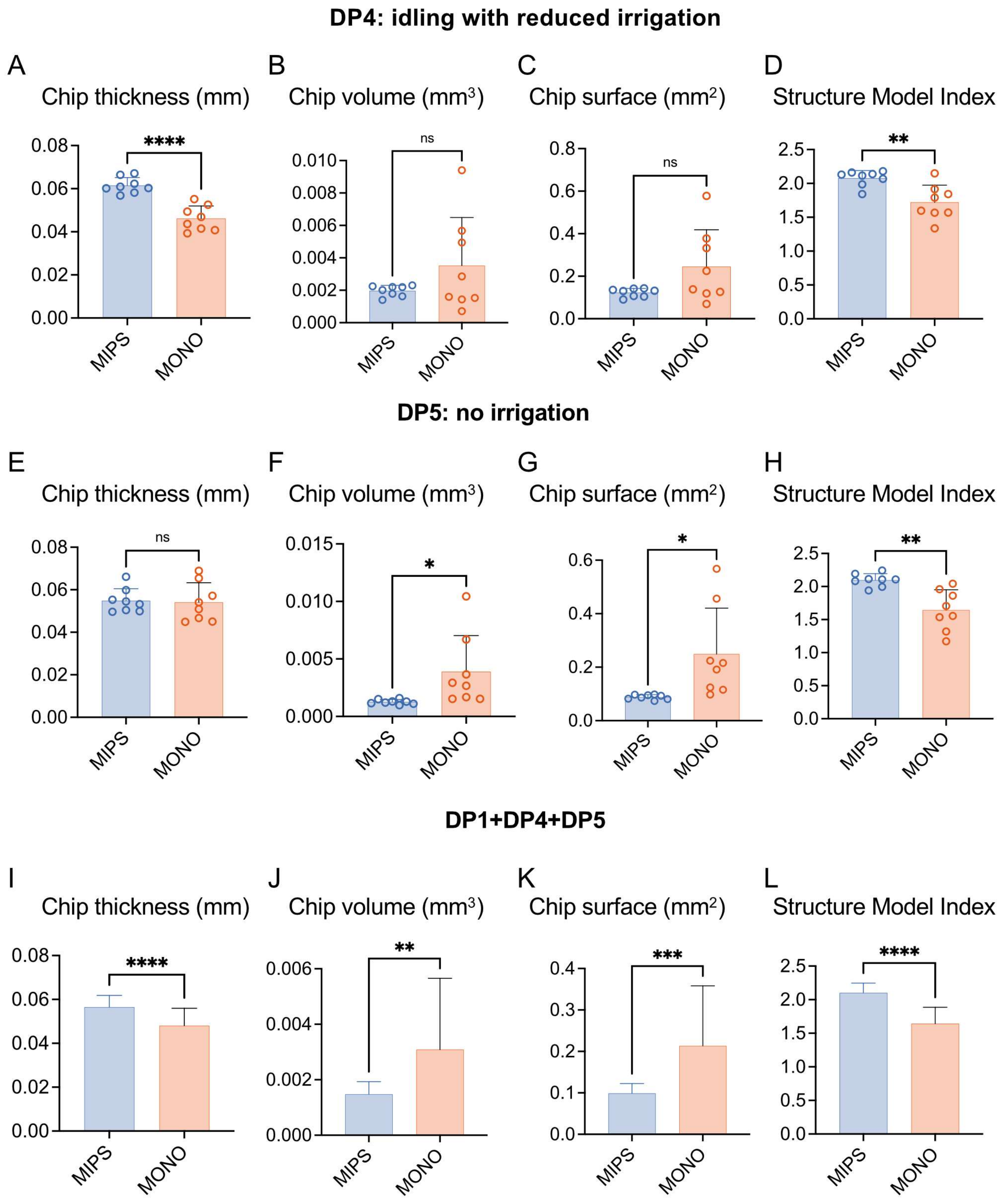

S3 Fig. Morphology of bone chips. Comparisons of the chip thickness, chip volume, chip surface area and structural model index obtained from microCT data quantification for (A-D) DP4, (E-H) DP5 and (I-L) DP+DP4+DP5 combined (parametric *t* test, $p < 0.05$). There, DP1-standard drilling, DP4-idling with reduced irrigation, and DP5-no irrigation.
